# Supplementary material for: Combined Fluorescence Fluctuation and Spectrofluorometric Measurements Reveal a Red-Shifted, Near-IR Emissive Photo-Isomerized Form of Cyanine 5
Source: Int J Mol Sci. 2023 Jan 19;24(3):1990. doi: 10.3390/ijms24031990 (PMC9916991; doi:10.3390/ijms24031990)
Supplement: Supplementary file 1 [file ijms-24-01990-s001.zip › ijms-2138687-supplementary.pdf]

Supplementary Materials:

Combined Fluorescence Fluctuation and Spectrofluorometric Measurements Reveal a Red-Shifted, Near-IR Emissive Photo-isomerized Form of Cyanine 5

Section S1. FCS curves recorded with excitation at different wavelengths

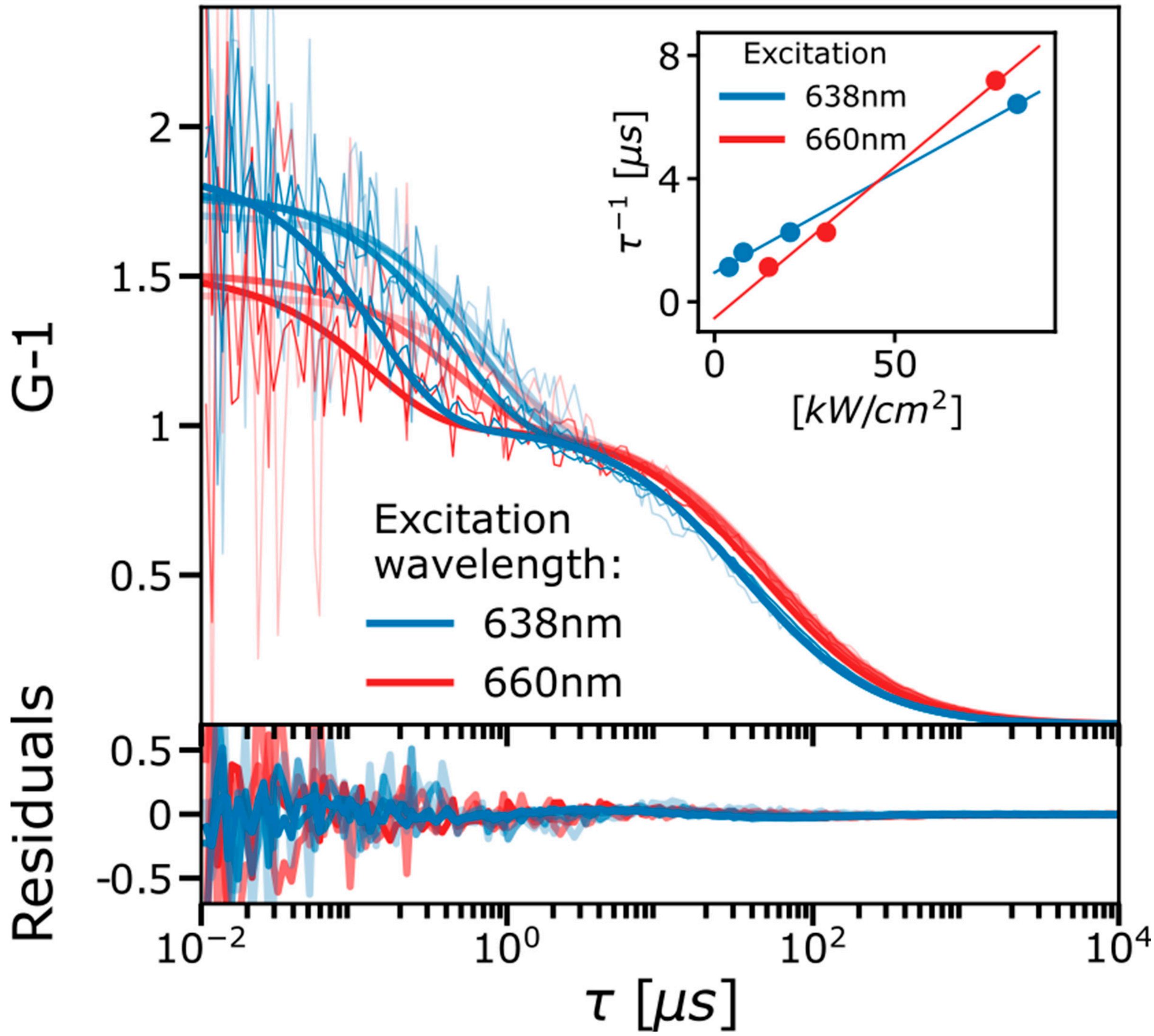

Figure S1. *FCS curves (thin lines) recorded at 638nm (blue) and 660nm excitation (red) with different* Φ_{exc} *applied, emission detected in the 690-750nm range. The FCS curves were fitted to a two-state trans-cis isomerization model (Eq. 8, thick solid lines, With the color-intensity representing increasing* Φ_{exc}*). Mean* Φ_{exc} *used: [15, 31, 78] kW/cm² for 660nm and [4, 8, 21, 84] kW/cm² for 638nm excitation. The inverse isomerization time (the isomerization relaxation rate) was found to be linearly dependent on the irradiance, as shown in the inset. The isomerization amplitudes remained constant, around 0.43 for 638nm excitation and 0.32 for 660nm excitation.*

Section S2. Fluorescence decay measurements of Cy5 by time-correlated single-photon counting (TCSPC)

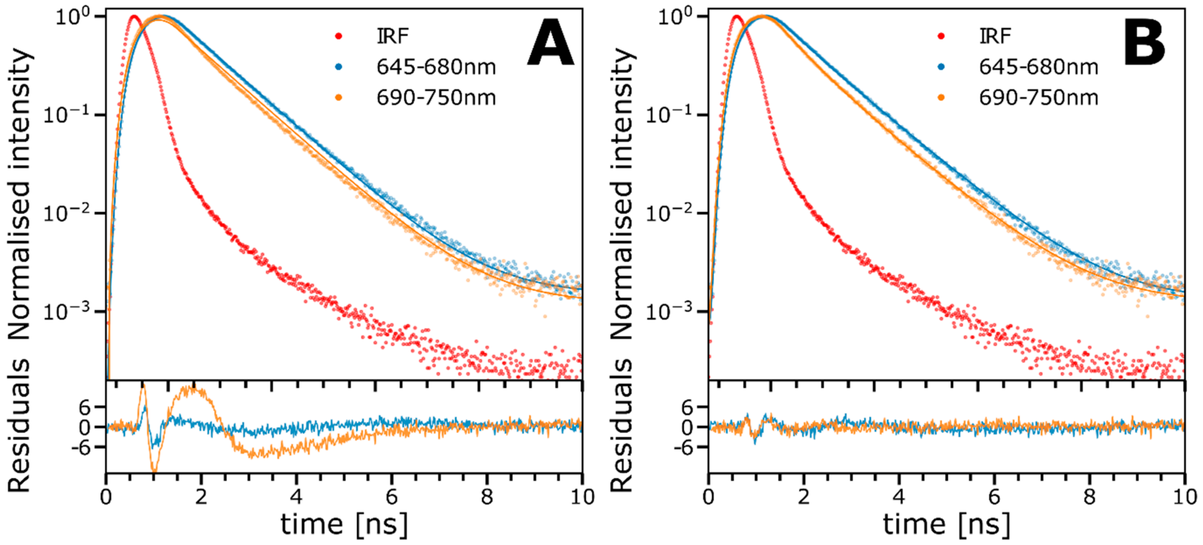

Figure S2. *TCSPC-measurements with 638nm excitation and with the fluorescence decay detected using different emission band pass filters (B-filter: 645-680nm, R-filter: 690-750nm). Fluorescence decay data, (dots) were deconvolved with the IRF (in red). Solid lines show fitted curves, with residuals below.*

(A): Data fitted with a mono-exponential decay. The decay model could be well fitted to the B-filter data, yielding a lifetime of τ_f*=1.0ns. The fit is worse with the R-filter used.*

(B): The same data as in (A), fitted with a bi-exponential decay. From the fitting residuals it can be seen that fitting the fluorescence decay measured through the R-filter with a bi-exponential decay led to a clearly better fit than when using a mono-exponential decay. For the B-filter data however, no major improvement was found when using a bi-exponential model. This provides supporting evidence that emission from an additional, more short-lived and red-shifted state is detected with the R-filter, but not with the B-filter. Resulting fitted parameter values: B-filter: τ_f*=1.0ns, R-filter:* τ_f*=1.0ns (fixed from (A)),* τ_{f2}*=0.5ns, with relative amplitude 0.38.*

Section S3. Electronic state model equations for Cy5

Similar to previous FCS studies of Cy5 isomerization kinetics [reference 32 in main text], the electronic state population dynamics of a Cy5 can be considered via a set of coupled linear differential equations, with the sum of the state populations normalized to 1. With the electronic state model shown in Figure 4B, the electronic state population dynamics of a Cy5 fluorophore, subject to a constant excitation photon flux of Φ_{exc} starting at time t=0, is given by

$$\frac{d}{dt}\bar{A}(t) = M \cdot \bar{A}(t) \qquad \text{(S1)}$$

Here, $\bar{A}(t) = [\,[N](t), [P_1](t),\ [P_2](t)]^T$ represents the population probabilities of the all-*trans*, a mono-*cis*, and a double-*cis* state and

$$M = \begin{bmatrix} -k_{iso1}' & k_{biso1}' & 0 \\ k_{iso1}' & -k_{biso1}' - k_{iso2}' & k_{biso2}' \\ 0 & k_{iso2}' & -k_{biso2}' \end{bmatrix} \qquad \text{(S2)}$$

is the model matrix describing the transitions between the states. In the matrix, the effective isomerization rates, from N to P_1, and from P_1 to P_2, are given by:

$$k_{iso1}' = k_{iso1} \cdot \frac{\sigma_N \cdot \Phi_{exc}}{\sigma_N \cdot \Phi_{exc} + k_{10}^N} \qquad \text{(S3A)}$$

$$k_{iso2}' = k_{iso2} \cdot \frac{\sigma_{P_1} \cdot \Phi_{exc}}{\sigma_{P_1} \cdot \Phi_{exc} + k_{10}^{P_1}} = \{k_{10}^{P_1} \gg \sigma_{P_1} \cdot \Phi_{exc}\} = \sigma_{iso2} \cdot \Phi_{exc} \qquad \text{(S3B)}$$

with σ_N and σ_{P_1} denoting the excitation cross sections of the singlet ground state of N and P_1, respectively, and with k_{10}^N and $k_{10}^{P_1}$ signifying the decay rates from the excited singlet state to the ground singlet state, in N and P_1 respectively. Since k_{iso2}, $k_{10}^{P_1}$ and σ_{P_1} could not be individually determined, we defined the isomerization from P_1 to P_2 as an isomerization cross section:

$$\sigma_{iso2} = k_{iso2} \cdot \frac{\sigma_{P_1}}{k_{10}^{P_1}} \qquad \text{(S3C)}$$

Similarly, the effective back-isomerization rates from P_1 to N, and from P_1 to P_2, are given by:

$$k_{biso1}' = k_{biso1} \cdot \frac{\sigma_{P_1} \cdot \Phi_{exc}}{\sigma_{P_1} \cdot \Phi_{exc} + k_{10}^{P_1}} + k_{biso1}^{Th} = \{k_{10}^{P_1} \gg \sigma_{P_1} \cdot \Phi_{exc}\} = \sigma_{biso1} \cdot \Phi_{exc} + k_{biso1}^{Th} \quad \text{(S4A)}$$

$$k_{biso2}{}' = k_{biso2} \cdot \frac{\sigma_{P_2} \cdot \Phi_{exc}}{\sigma_{P_2} \cdot \Phi_{exc} + k_{10}^{P_2}} + k_{biso2}^{Th} = \{k_{10}^{P_2} \gg \sigma_{P_2} \cdot \Phi_{exc}\} = \sigma_{biso2} \cdot \Phi_{exc} + k_{biso2}^{Th} \quad \text{(S4B)}$$

With corresponding back-isomerization cross sections defined as:

$$\sigma_{biso1} = k_{biso1} \cdot \frac{\sigma_{P_1}}{k_{10}^{P_1}} \quad \text{(S4C)}$$

$$\sigma_{biso2} = k_{biso2} \cdot \frac{\sigma_{P_2}}{k_{10}^{P_2}} \quad \text{(S4D)}$$

The initial condition for Eq. (S1) is

$$\bar{A}(0) = [1\ 0\ 0\ ]^T \quad \text{(S5)}$$

, assuming all Cy5 fluorophores are in the singlet (ground) state before onset of excitation at $t = 0$.

For a rectangular excitation pulse, Φ_{exc} is constant throughout the excitation duration and the matrix M is not time dependent. The general solution to Eq. S1 is then

$$\bar{A}(t) = e^{Mt} \cdot \bar{A}(0) \quad \text{(S6)}$$

The dependence of the detected fluorescence at time, *t*, after onset of excitation is then given by

$$F(t) = {}^1q_F \cdot {}^1q_D \cdot k_{10}^N \cdot \frac{\sigma_N \cdot \Phi_{exc}}{\sigma_N \cdot \Phi_{exc} + k_{10}^N} \cdot [N](t) + {}^2q_F \cdot {}^2q_D \cdot k_{10}^{P_2} \cdot \frac{\sigma_{P_2} \cdot \Phi_{exc}}{\sigma_{P_2} \cdot \Phi_{exc} + k_{10}^{P_2}} \cdot [P_2](t) \quad \text{(S7)}$$

, with σ_X denoting the excitation cross section, ${}^Xq_F$ the fluorescence quantum yield and ${}^Xq_D$ the detection quantum yield of the emission from N (X=1) and P_2 (X=2) state, respectively. For the excitation conditions in our study, $k_{10} \gg \sigma_N \cdot \Phi_{exc}, \sigma_{P_2} \cdot \Phi_{exc}$, so that we can assume

$$F(t) = {}^1q_F \cdot {}^1q_D \cdot \sigma_N \cdot \Phi_{exc} \cdot ([N](t) + Q \cdot [P_2](t)) \quad \text{(S8)}$$

, with $Q = ({}^2q_F \cdot {}^2q_D \cdot \sigma_\mathrm{N})/({}^1q_F \cdot {}^1q_D \cdot \sigma_{P_2})$ representing the relative brightness of N, compared to P_2.

Section S4: TRAST and FCS data fitted to a two-state photo-isomerization model

As an alternative model to the three-state model of Figure 4B, the experimental FCS and TRAST data of Figures 1-3 were also fitted to a two-state photo-isomerization model, with P_1 and P_2 viewed as one single mono-cis state with red-shifted fluorescence emission. In such two-state model, with P_1 and P_2 merged into one photo-isomerized state, P, the merged P state may either indeed represent a single, weakly fluorescent mono-*cis* state, or a time-average of one or several photo-isomerized forms and a double-photo-isomerized form of Cy5 (red-marked in Figure 4B). In this case, the isomerization rates $k_{biso1}{}'$, $k_{iso1}{}'$ were defined as for the three-state model (Eqs. S3A and S4A) and fitted by the same procedure, while the rates and cross-sections for the transitions between P_1 and P_2 were not included. For the two-state model, the initial condition used in the fitting of the FCS curves was redefined as:

$$[N](\bar{r},0) = \frac{\bar{N}(\bar{r})}{\bar{N}(\bar{r})+Q\bar{P}_2\ (\bar{r})}$$

$$[P_1\ ](\bar{r},0) = \frac{Q\bar{P}_1\ (\bar{r})}{\bar{N}(\bar{r})+Q\bar{P}_1\ (\bar{r})} \qquad \text{(S10)}$$

With both sets of FCS curves in Figure 2 globally fitted to Eq. 5, and with $G_T(\tau)$ defined by a two-state model (Eq. 10, with P_2 replaced with P) could well reproduce the experimental curves, and yielded the following parameter values: k_{iso}=32μs^{-1}, $\sigma_{biso1} = 0.12 \cdot 10^{-16}$cm^2 and $Q = 0.23$, (See Figure S3A).

Both sets of TRAST curves (Figure 3), recorded by the B- and R-emission filter were likewise jointly fitted with global parameters to the same two-state model, by otherwise using the same procedure as for the three-state model. The fitting resulted in curves which could well reproduce the experimental TRAST curves and yielded the following parameter values: k_{iso}=32μs^{-1}, $\sigma_{biso1} = 0.12 \cdot 10^{-16}$cm^2, k_{th1}=0.02μs^{-1}and Q=0.14 (for the TRAST curves recorded with the R-emission filter), (See Figure S3B). Thus, fitted parameter values could be obtained also for the two-state model, which were in good agreement between the FCS and TRAST data.

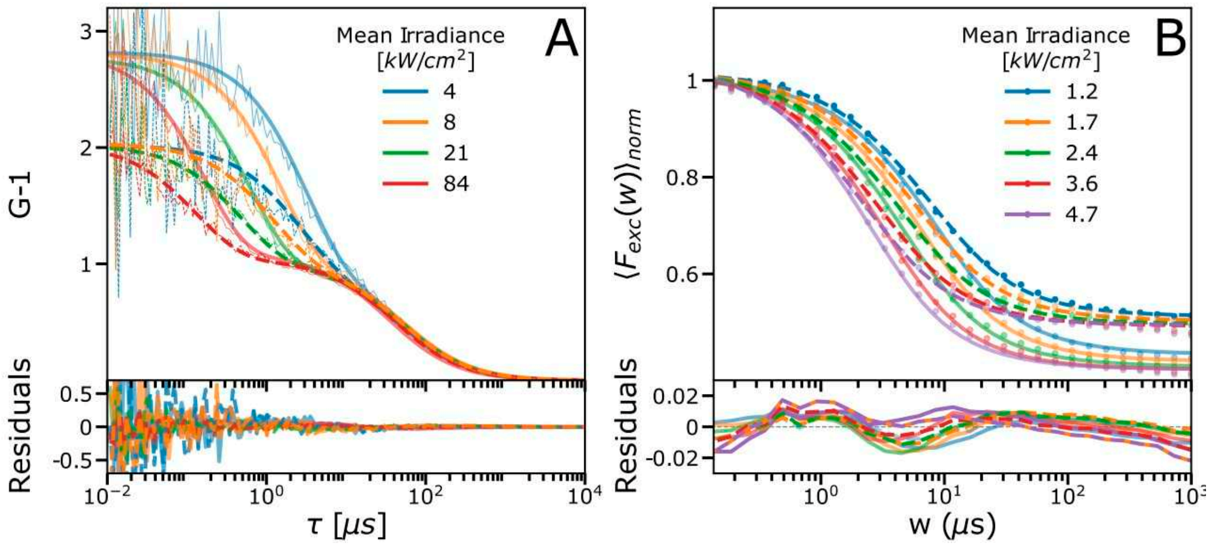

Figure S3: *FCS and TRAST curves fitted to a two-state photo-isomerizaiton model.*

(A): FCS curves (the same experimental curves as in Figure 2), recorded under different mean Φ_{exc} *(638nm excitation) and using two different emission filters: 645-680nm (B-filter, data: thin solid lines, fitted curves: thick lines) and 690-750nm (R-filter, data: dotted lines, fit: thick dotted lines). The FCS curves were fitted globally to a two-state model as described in the text above. Fitting residuals are shown in the lower subplot.*

(B): TRAST-curves recorded under different mean Φ_{exc} *(638nm excitation) and using two different emission filters: 645-680nm (B-filter, data: thin solid lines, fitted curves: thick lines) and 690-750nm (R-filter, data: dotted lines, fit: thick dotted lines). The TRAST curves were globally fitted, as described in the text above. Fitting residuals are shown in the lower subplot.*

Section S5. Spatial distribution of excitation rates, calculation of average rates in the TRAST experiments

The excitation beam does not form a uniform profile, and the excitation photon flux, $\Phi_{exc}(\bar{r})$, is thus a function of position in the sample. As a consequence, our TRAST analysis included a spatial dependence on both the excitation rates and the resulting electronic state populations. The total fluorescence signal on each pixel of the camera then becomes a convolution of $[N](t) + Q \cdot [P_2](t)$ and the microscope collection efficiency function, $CEF(\bar{r})$. While simulating the whole 3D sample volume, and computing the projected 2D image on the camera, we found that pre-computing an average observed excitation rate, $\hat{k}_{01}$, for each ROI to be analyzed, speeds up the fitting significantly, without appreciable loss of accuracy. The approximate $\hat{k}_{01}$ is computed once, before fitting starts, by weighting $k_{01}(\bar{r})$ by brightness and collection efficiency, $CEF(\bar{r})$, in the following manner

$$\hat{k}_{01} = \frac{\iiint k_{01}(\bar{r}) \cdot \hat{S}_1(\bar{r}) \cdot CEF(\bar{r})\, dV}{\iiint \hat{S}_1(\bar{r}) \cdot CEF(\bar{r})\, dV} \qquad (S9)$$

$\hat{S}_1(\bar{r}) = k_{01}(\bar{r})/(k_{10} + k_{01}(\bar{r}))$ represents the population of excited singlet state Cy7 when in an all-*trans* form, N, at onset of excitation, after equilibration between the ground and excited singlet states of N, but before build-up of the other states.
